# Partitioning the Sleep Quality and Insomnia Severity among Earthquake Victims in the West of Iran: Cluster Prediction Based on Personality and Psychological Factors

**Published:** 2019-09-08

**Authors:** Habibolah Khazaie, Farid Najafi, Ali Zakiei, Saeid Komasi

**Affiliations:** ^1^Sleep Disorders Research Center, Kermanshah University of Medical Sciences, Kermanshah, Iran; ^2^Research Center for Environmental Determinants of Health, Kermanshah University of Medical Sciences, Kermanshah, Iran; ^3^Clinical Research Development Center, Imam Reza Hospital, Kermanshah University of Medical Sciences, Kermanshah, Iran

**Keywords:** Cluster analysis, Earthquake, Emotion, Insomnia, Sleep quality

## Abstract

**Background:** We aimed to perform a cluster analysis on sleep quality and insomnia severity in addition to predicting the clusters based on personality traits, experiential avoidance, stress, anxiety, depression, and dysfunctional beliefs and attitudes about sleep.

**Study Design:** A cross-sectional study.

**Methods:** This study was conducted on earthquake victim in Kermanshah (western Iran) in 2017. Data collection began 15 d after the earthquake and lasted for 2 weeks. First, 1002 copies of the questionnaire were distributed and, finally, analyses were performed for 778 individuals. Data analysis was conducted using cluster analysis.

**Results:** Based on sleep quality and insomnia severity, four clusters were formed, and a correlation existed between some personality traits, psychological distress, experiential avoidance, and dysfunctional beliefs and attitudes about sleep with unhealthy clusters (P<0.05). The summary of the model showed the sufficient fit of the model (P<0.001) and that it could predict 22.8%-42.4% of the variance of unhealthy clusters.

**Conclusion:** Dysfunctional beliefs and attitudes about sleep, experiential avoidance, stress, anxiety, depression, and personality traits could contribute to sleep problems and reduce sleep quality in earthquake victims.

## Introduction

Crises, natural or social, always have outcomes affecting the social, family, and psychological life in general. Of natural disasters, earthquakes are among the most unpredictable and deadly ones with destructive economic and social effects^1^. In addition to mortality and physical injury, earthquakes can cause psychological-behavioral problems in both injured persons and survivors, disrupting their ability to perform social and economic activities for a long time^2^.Sleep problems are among these issues^3^. In these conditions, the prediction of sleep quality and its impressionability by the earthquake is a significant topic which merits further studies.

Studies examining the role of cognitive factors in sleep have emphasized the role of pre-sleep and night-time cognitions related to sleep ^4, 5^. Dysfunctional beliefs and attitudes about sleep are considered as a predictor of sleep profile. In addition, personality traits play a key role in any behavior, including sleep. Personality traits are among the predictors of sleep quality^6-8^. Personality traits are able to identify the clusters where there is a significant difference in regard to sleep quality and the intensity of insomnia^9^.

Results of studies suggest the role of emotions in sleep disorders ^4, 10^. Moreover, emotional situations (e.g. earthquakes and their aftermath) may variably affect sleep patterns ^10, 11^. Therefore, it is important to study stress, depression, and anxiety as predictor variables. A review of studies on the effect of emotions on sleep indicates that, in all these studies, the main focus is on arousal. In fact, arousal has various effects on a range of sleep parameters. The operational definitions of arousal are not the same in all these studies. Nevertheless, all of them have considered arousal a multi-faceted construct encompassing thoughts, physical sensations, and stressful behaviors all of which have facets of emotional arousal^12^. Evidence suggests a mutual, complex relationship between emotion and sleep^13^.

In recent studies, the role of experiential acceptance and avoidance in sleep problems has been confirmed. Experiential avoidance can be part of psychological flexibility. Psychological flexibility can be a strong predictor of insomnia severity, sleep problems, low sleep efficiency, and fatigue^14^. The number of avoidance behaviors is directly related to sleep disturbances^15^.

We aimed to identify the subgroups of Kermanshah earthquake survivors based on the factors affecting these partitions using cluster analysis. Currently, a lot of data are available to psychologists and psychotherapists considered worthless because of poor knowledge related to its application and the data are neglected. However, if the same data are purposefully stored and ultimately mined, it will produce a great deal of knowledge and help us make psychological decisions. Therefore, data mining techniques such as clustering can be used and the hidden patterns can be extracted from the existing data to help professionals in making the optimal and timely decisions on psychological issues. This is more valuable in crises such as earthquakes because crisis intervention requires short-term, yet useful, solutions.

The examination of the role of personality traits in the prediction of sleep profile enhances our understanding and knowledge of the effect of personality on sleep. On the other hand, attention to emotions such as anxiety, depression, and stress, especially in conditions like an earthquake, helps in the provision of psychological services. Thus, we aimed to perform a cluster analysis on sleep quality and insomnia severity and predict clusters based on personality traits, experiential avoidance, anxiety, depression, and dysfunctional beliefs and attitudes about sleep.

## Methods

This cross-sectional study was conducted among earthquake victims in urban and rural areas of Kermanshah Province (Western Iran) in 2017. We decided to conduct this study after the earthquake with a magnitude of 7.3 on Oct 2017, which killed and injured many and destroyed at least three cities. Data collection began 10 d following the earthquake and continued for two weeks. First, 1002 copies of the questionnaire were distributed among residents of Sarpol-e Zahab (epicenter). Eventually, 778 copies were returned and analyzed. Participation was voluntary and with the participants’ full consent. Copies of the questionnaire were given to those aging at least 15 years who could read and write. They were also local residents with a minimum of 5 years’ history of residence there. Responding to items was performed in the presence of a research team member who provided the required directions.

After adjusting the questionnaires and selecting the subjects, the samples responded to the research questionnaires. Then, the researcher was provided the necessary explanations on how to complete the questionnaires. Subjects were asked to seek help from the researcher if they had challenge in answering questions. In order to adhere to the research ethics, individuals were asked to withdraw from the study if they did not consent to participate. Selected individuals completed the questionnaires after consenting to participate in the research and receiving assurances of confidentiality. After completing the questionnaires, the papers were collected by the research team.

Although no funds were provided to the participants by the research team, safety of each subject during and after the research was the top priority of the research team. The project was designed and implemented by people with the necessary skills and related expertise. Therefore, this study did not lead to any health and financial risk to the participants. Exclusion criteria were included unwillingness to participate in the study, serious earthquake-related injury including amputation and spinal cord injury, having chronic physical illnesses such as pulmonary problems, non-natives, and peoples less than 15 yr of age.

This study was registered at the Sleep Disorders Research Center of Kermanshah University of Medical Sciences in Iran and was approved by the Ethics Committee of the university (IR.KUMS.REC.1396.561).

### Data Collection Instrument

*1. The Pittsburgh Sleep Quality Index (PSQI):* PSQI is a self-report questionnaire designed by Buysse et al. This questionnaire is a standard 18-item scale in which items are categorized under 7 components. The first component is subjective sleep quality measured using one item (Item 9). The second component is sleep latency, measured with two items, i.e. the mean of Item 2 score and the score of Part "A" of Item 5. The third component is sleep duration, examined using one item (Item 4). The fourth component is habitual sleep efficiency whose total score is calculated by dividing the total hours of sleep on the hours the person lies in bed, multiplied by 100. The fifth component is sleep disturbances measured by calculating the mean of scores to Item 5. The sixth component is the use of sleeping medication, examined using one item (Item 6). The seventh component is daytime dysfunction measured using two items (mean of scores of Items 7 and 8). The score of each item ranges from 0 to 3, and the maximum score for each component is 3. These seven components comprise the total score of the scale, ranging from 0 to 21. The higher the total score, the lower the sleep quality. Scores above 5 indicate a fairly bad sleep quality. In a study, the reliability of this instrument was examined that Cronbach’s alpha in total sample was 0.78, in clinical sample was 0.81 and in the non-clinical sample was 0.71^16^. This value was 0.94 in the present study.

*2. Insomnia Severity Index (ISI):* This 7-item questionnaire was employed to measure the severity of insomnia. It includes items to evaluate problems with sleep onset, sleep maintenance, early morning wakening problems, sleep dissatisfaction, interference of sleep difficulties with daytime functioning, effects of sleep disturbance on the quality of life, and distress caused by the sleep difficulties. Each item receives a score of 0 to 4 depending on severity, and the total score is determined by adding the scores of these seven items^17^. In this study, the reliability of this instrument was examined that Cronbach’s alpha, this value was 0.92.

*3. Zuckerman-Kuhlman Personality Questionnaire (ZKPQ-50):* The 50-item version of ZKPQ was employed here. It includes five personality factors based on Zuckerman's five-factor model for personality. These five factors are neuroticism-anxiety, impulsive sensation seeking, sociability aggression-hostility, and activity. Each factor has 10 items. The respondent provides his/her answer for each item on a binary scale (true/false). In some cases, true receives the score of 1 and false receives 0. In others, the reverse is true^18^. One study reported Cronbach's alpha of 0.70 for this questionnaire^19^. This value was 0.86 for the entire questionnaire in the present study.

*4. The Acceptance and Action Questionnaire (AAQ):* AAQ was developed by Hayes et al. Its first version included 32 items scored on a 7-point Likert scale. Following versions included 16 and 9 items, but the last version of this questionnaire used in the present study has 10 items scored on a 7-point Likert scale. One study reported a one-factor structure for this scale with the Cronbach’s alpha of 0.84^20^. The reliability of this questionnaire was examined in Iran in 2012 with the Cronbach’s alpha of 0.82. The Beck Anxiety Inventory, Beck Depression Inventory, and the Regulation of Emotions Questionnaire have been used to evaluate the validity of this questionnaire, with correlation coefficients of 0.44, 0.59, and 0.59. Results of factor analysis showed the appropriate weight of factors^21^. Cronbach’s alpha for this questionnaire equaled 0.76 in the present study. The score of this questionnaire indicates the level of experiential avoidance in individuals and higher score means more experiential avoidance.

*5. Dysfunctional Beliefs and Attitudes about Sleep Scale (DBAS):* This scale was developed by Morin, including 10 items related to dysfunctional beliefs and attitudes before sleep which play a role in sleep problems. Respondents must indicate their agreement with each item on a Likert scale ranging from 0 to 10. Higher scores indicate a higher level of dysfunctional attitudes and beliefs about sleep. The internal consistency of this scale (Cronbach’s alpha) was reported to be 0.77 and 0.79 for clinical and normal populations, respectively ^22^. Cronbach’s alpha for this questionnaire equaled 0.93 in the present study.

*6. The Depression Anxiety Stress Scales (DASS):* DASS was developed by Lovibond and Lovibond. This scale has 21 items, each with four options: “does not apply to me at all” (0), “applied to me to some degree, or some of the time” (1), “applied to me to a considerable degree, or a good part of time” (2), and “applied to me very much, or most of the time” (3). Of the 21 items in this questionnaire, each factor has 7 items. A factor analysis of this questionnaire was performed, and results indicated the existence of three factors (anxiety, stress, and depression). Alpha coefficient equaled 0.97, 0.92, and 0.95 for the noted factors^23^. This questionnaire was examined in Iran, with the test-retest validity of 0.80, 0.76, and 0.77 for depression, anxiety, and stress scales, and the alpha of 0.81, 0.74, and 0.78, respectively^24^.

### Statistical Analysis

Data belonging to continuous variables are reported as mean and SD, and discrete data are reported as percentage. In order to identify the number of clusters, first, the hierarchical centroid clustering method with squared Euclidean distance was used. The centroid clustering method was employed to prevent the extensive effect of multiple completely related variables (sleep components) on one another. Models including 2 to 10 clusters were separately evaluated, and the discriminative power of the four-cluster model is the highest; Because the distribution of the samples in these clusters was more than two individuals in each the cluster. In the next step, the k-means clustering method was utilized to determine the 4 clusters proposed by the initial model. Finally, Cramér's V was used to examine the stability of the cluster solution structure and determine the concordance among solutions. Sleep components in clusters were compared using ANOVA.

In the following step, the relationship between clusters and demographic and psychological factors was examined using multinomial logistic regression. In this analysis, Cluster 2 (the population with good sleep and without insomnia) was considered the reference. All psychological variables (personality traits, experiential avoidance, anxiety, stress, depression, and dysfunctional beliefs about sleep) and demographic factors were simultaneously entered into the model. Results of the analysis are reported in terms of adjusted odds ratios (OR) with 95% confidence intervals (CIs). All statistical analyses were performed in SPSS 20 (IBM Corp., Armonk, NY, USA). All tests were two-tailed and statistical significance was defined as *P*<0.05.

## Results

Results for 778 individuals (aging 15-68 yr) were analyzed, of which 57% were female. Mean age of participants was 30.86±11.23 year.

Table 1 shows the profile resulting from hierarchical and k-means cluster analysis. Results of Cramer's V (0.37, *P*=0.0005) showed that the cluster solution structure in both models had appropriate stability and a concordance was observed between solutions. This model proposed 4 clusters for sleep components. A significant difference existed among clusters in all components (*P*<0.0005). The proposed clusters are (i) population with very low sleep quality and severe insomnia, (ii) population with good sleep quality and without insomnia, (iii) population with relatively low sleep quality and mild insomnia, and (iv) population with low sleep quality and moderate insomnia.

**Table 1:** The profile derived from a cluster analysis (n = 778)

**Variable**s	**Total** **n=778**		**Cluster 1** ^a^ **n=31**		**Cluster 2** ^b^ **n=488**		**Cluster 3** ^c^ **n=171**		**Cluster 4** ^d^ **n=88**		***P*** **value**
	**Mean**	**SD**	**Mean**	**SD**	**Mean**	**SD**	**Mean**	**SD**	**Mean**	**SD**	
Pittsburgh Sleep Quality											
Subjective sleep quality	1.14	0.58	2.20	0.81	0.94	0.34	1.13	0.46	1.88	0.72	0.001
Sleep latency	0.97	0.74	2.06	0.69	0.72	0.60	1.13	0.64	1.64	0.80	0.001
Sleep duration	0.57	0.59	0.96	0.95	0.52	0.52	0.58	0.56	0.73	0.75	0.001
Sleep disturbance	0.80	0.62	1.80	0.59	0.56	0.51	0.97	0.39	1.47	0.58	0.001
Use of sleep medication	0.22	0.59	1.12	1.12	0.05	0.23	0.24	0.49	0.84	1.03	0.001
Daytime dysfunction	0.45	0.69	1.66	0.74	0.17	0.40	0.66	0.63	1.20	0.85	0.001
Sleep efficiency	0.41	0.70	0.54	0.75	0.31	0.62	0.59	0.79	0.53	0.83	0.001
Insomnia Severity Index	5.92	5.15	20.88	2.34	2.72	1.45	8.05	1.42	14.27	1.73	0.001

^a^ Population with a very poor quality sleep and severity insomnia. All values are as mean ± standard deviation ^b^ Population with a good quality sleep without insomnia ^c^Population with a relatively poor quality sleep and mild insomnia ^d^Population with a poor quality sleep and moderate insomnia

Table 2 presents the demographic and psychological information of each cluster. Moreover, this table provides the results of the multinomial logistic regression analysis. Based on this table, some demographic components (age, level of education, and occupation) significantly correlated with unhealthy clusters (*P*<0.05). In other words, younger individuals and those with a lower level of education have a lower risk of being placed in unhealthy clusters. Conversely, unemployed individuals and university students have a higher risk of being placed in unhealthy clusters. In terms of psychological components, correlations exist between unhealthy clusters and some personality traits, depression, anxiety, stress, experiential avoidance, and beliefs about sleep (*P*<0.05). Unhealthy clusters have a higher degree of neuroticism-anxiety and lower level of sensation seeking. Each unit increases in the score for neuroticism and reduces in the sense-seeking score of 0.7 to 0.8 times reduces sleep quality. Conversely, the aggression attribute score in the reference cluster is higher. The level of depression, stress, dysfunctional beliefs about sleep, and experiential avoidance is also significantly higher in unhealthy clusters compared to the reference cluster. These components reduce the quality of sleep by 1.6 times. The summary of the model shows the sufficient fit of the model (*P*<0.0005) and that it can predict 22.8%-42.4% of the variance of unhealthy clusters (Table 2).

**Table 2:** The results of multinomial regression logistic for identifying correlates

**Variables** **Categorical variables**	**Cluster 1** ^a^ **, n=31**				**Cluster 2** ^b^ **(Ref.). n=488**		**Cluster 3** ^c^ **, n=171**				**Cluster 4** ^d^ **, n=88**			
	**Number**	**Percent**	**OR**	**95% CI**	**Number**	**Percent**	**Number**	**Percent**	**OR**	**95% CI**	**Number**	**Percent**	**OR**	**95% CI**
Sex, female	18	58.1	0.57	0.20, 1.68	252	51.6	93	54.4	0.90	0.59, 1.36	53	60.2	0.98	0.56, 1.73
Age group (yr)														
<19	2	6.5	0.05	0.01,1.88	89	18.2	32	18.7	0.35	0.08, 1.52	4	4.5	**0.05**	0.01, 0.48
20-44	21	67.7	**0.04**	0.01, 0.53	334	68.4	116	67.8	0.35	0.09, 1.31	63	71.6	**0.17**	0.03, 0.95
45-64	7	22.6	0.22	0.01, 3.65	60	12.3	17	9.9	0.33	0.08, 1.36	18	20.5	0.60	0.10, 3.70
>64	1	3.2	Ref.		5	1.0	6	3.5	Ref.		3	3.4	Ref.	
Marriage status														
Marriage	22	71	0.72	0.10, 5.15	266	54.5	85	49.7	0.71	0.25, 2.05	61	69.3	2.96	0.58, 15.08
Single	6	19.4	0.12	0.01, 1.12	210	43.0	79	46.2	0.82	0.27, 2.44	25	28.4	1.98	0.36, 10.77
Widow/divorce	3	9.7	Ref.		12	2.5	7	4.1	Ref.		2	2.3	Ref.	
Education level														
Elementary	11	35.5	**0.15**	0.03, 0.79	283	58.0	90	52.6	0.77	0.39, 1.50	46	52.3	0.55	0.22, 1.40
Under diploma	4	12.9	0.60	0.08, 4.67	55	11.3	17	9.9	0.62	0.27, 1.43	10	11.4	1.08	0.35, 3.39
Diploma	7	22.6	0.37	0.07, 1.96	99	20.3	37	21.6	0.88	0.44, 1.77	21	23.9	0.74	0.28, 1.94
Academic	9	29.0	Ref.		51	10.5	27	15.8	Ref.		11	12.5	Ref.	
Job type														
Unemployed	15	48.4	**7.93**	1.85, 33.94	239	49.0	87	50.9	1.43	0.83, 2.46	55	62.5	**2.26**	1.11, 4.62
Student	5	16.1	**14.58**	1.31,162.65	85	17.4	35	20.5	1.46	0.66, 3.24	9	10.2	1.93	0.55, 6.75
Clerk	3	9.7	1.64	0.09, 31.50	12	2.5	6	3.5	1.51	0.43, 5.29	1	1.1	0.22	0.02, 2.38
Farmer	0	0.0	2.66	NA	38	7.8	11	6.4	1.35	0.58, 3.15	0	0.0	2.49	NA
Self-employed	8	25.8	Ref.		114	23.4	32	18.7	Ref.		23	26.1	Ref.	
**Categorical variables**	Mean	SD	OR	95% CI	Mean	SD	Mean	SD	OR	95% CI	Mean	SD	OR	95% CI
Personality traits														
Neuroticism	15.7	2.2	**0.68**	0.52, 0.90	14.5	2.5	14.8	2.3	1.07	0.97, 1.18	15.3	2.4	0.95	0.83, 1.08
Sense-seeking	11.4	2.3	0.94	0.74, 1.19	11.2	2.2	10.5	1.8	**0.84**	0.75, 0.93	11.0	2.3	0.88	0.76, 1.00
Socializing	12.1	1.9	1.12	0.84, 1.49	12.4	1.6	12.2	1.6	0.98	0.87, 1.10	12.1	1.8	1.00	0.86, 1.17
Activity	9.6	1.5	1.33	0.90, 1.99	9.2	1.3	9.3	1.4	1.03	0.90, 1.19	9.2	1.3	0.99	0.81, 1.20
Aggression-hostility	13.7	2.2	1.07	0.82, 1.38	13.5	1.9	12.8	1.8	**0.87**	0.78, 0.96	13.4	2.2	0.99	0.86, 1.13
DASS														
Depression	13.3	4.0	**0.80**	0.66, 0.99	8.7	5.1	8.7	3.9	0.98	0.91, 1.07	10.9	4.3	**0.83**	0.75, 0.92
Anxiety	13.9	4.2	1.13	0.92, 1.38	8.0	4.8	7.6	3.7	0.86	0.79, 0.94	10.7	4.4	0.90	0.81, 1.00
Stress	15.3	3.5	**1.79**	1.41, 2.29	8.4	4.5	8.9	3.7	**1.18**	1.06, 1.30	12.3	3.8	**1.56**	1.37, 1.78
Sleep beliefs	69.4	24.6	**1.08**	1.05, 1.11	49.6	16.6	58.7	16.9	**1.03**	1.02, 1.05	57.4	18.4	**1.03**	1.02, 1.05
Experiential avoidance	43.6	6.7	**1.28**	1.16, 1.41	34.4	7.9	35.1	5.8	1.03	0.99, 1.06	39.5	7.1	**1.10**	1.06, 1.16

^a^ Population with a very poor quality sleep and severity insomnia. All values are as mean ± standard deviation ^b^ Population with a good quality sleep without insomnia ^c^Population with a relatively poor quality sleep and mild insomnia ^d^Population with a poor quality sleep and moderate insomnia Summary of model: The model fitting information is: Chi-square = 355.651, *P*<0.0005; Pseudo R-square based on McFadden and Nagelkerke = 0.228 to 0.424; Boldface indicates statistically significant (*P*<0.05)

## Discussion

Results of the present study revealed that, based on sleep quality and insomnia severity, four clusters are formed: (i) population with very low sleep quality and severe insomnia, (ii) population with good sleep quality and without insomnia, (iii) population with a relatively low sleep quality and mild insomnia, and (iv) population with low sleep quality and moderate insomnia. Results of the analysis for predicting these clusters revealed that a correlation exists between unhealthy clusters and some personality traits (including neuroticism), psychological distress (stress, anxiety, and depression), experiential avoidance, and dysfunctional beliefs and attitudes about sleep. This model has a good fit and can predict 22.8%-42.4% of the variance of unhealthy clusters. In the literature review, no similar study performing such cluster analysis was found^6-8, 25^. Emotional situations (conditions such as an earthquake) may variably affect sleep patterns, or emotional problems can lead to issues in falling asleep or sleep disturbances^11^. A correlation existed between depressive mood and anxiety on the one hand, and sleep quality on the other, through rumination^26^. In this study, the role of cognitive factors, i.e. negative repeated thoughts, examined. Moreover, in this study, emotional factors such as depressive mood and anxiety have been investigated which is, a combination of cognitive and emotional factors. Results of the noted study are somewhat consistent with those of the present study.

Stress, anxiety, and depression were higher in those with a lower-quality sleep than those with an optimal-quality sleep^27^. In general, those with mood disorders (such as depression) and anxiety usually had a lower sleep quality^28^. Anxious states were higher in those with a lower-quality sleep,^29^ which is consistent with our findings. In addition, the association between stresses and sleep quality has been confirmed and therefore stress is considered as an important factor in the etiology of primary insomnia.

Anxiety and stress could be predisposing factors for insomnia^30^. As cognition, emotion, and behavior interact in affecting sleep, we believe that those who have the problem in regulating the level of stress and anxiety are more likely to be impressed by anxiety and, therefore, have a lower quality of sleep. On the other hand, the major cause of insomnia is arousal. It seems that stress acts as an arousing factor ^5, 31^. Therefore, sleep quality is reduced in those with a higher level of stress and anxiety.

Based on acceptance and commitment therapy, experiential avoidance leads to attempts for suppressing emotions, thoughts, and other internal experiences. This psychological inflexibility leads to emotional disorders^32^. Experiential avoidance leads to numerous forms of psychological pathology, including drug abuse, panic attack, borderline personality disorder, post-traumatic stress disorder^33^. Meanwhile, research has shown a relationship between emotional disorders and sleep disorders^34^. Therefore, experiential avoidance can be related to insomnia as well. Furthermore, the avoidance of internal thoughts and experiences increases physiologic arousal and negative emotions^35^. This arousal leads to insomnia and reduced sleep quality.

Furthermore, those with neuroticism have a strong inclination towards experiencing stress^36^ and have problems with emotion regulation^37^. Thus, they are predisposed to insomnia and reduced sleep quality. On the other hand, those with neuroticism are sensitive to signs of threatening and dysfunction which may cause their poor performance in response to sleep disturbance perception^8^. This is more tangible in stressful situations like those experienced by our sample. These people may find such situations more threatening than other people do and, thus, have a poor performance in the face of a destroyed bedroom and having to sleep in a shelter or tent.

Dysfunctional beliefs and attitudes about sleep play an important mediating role in the continuation of insomnia^30^. In our sample, participants are in stressful conditions. If they have dysfunctional beliefs and attitudes about sleep, they face more sleep problems in this condition considering the mediating role of these beliefs. To explain these results, one can refer to Espie's theory^30^. Cognitive-behavior theories are based on the concept that cognition, emotion, and behavior interact with one another, and anomalous thoughts can cause negative emotions, leading to changes in behavior^30^.

Results of the present study also showed that about 4% of the sample had a very poor sleep quality and severe insomnia, over 100% belonged to the cluster of poor sleep quality and moderate insomnia, and over 22% had a relatively poor sleep quality and mild insomnia. This shows that the sleep quality of this sample is not optimal. These results are in line with those of previous studies ^3, 38^ reporting that individuals experience reduced sleep quality, increased insomnia, and other sleep disorders following an earthquake. Earthquake victims do not have desirable psychological conditions due to the destruction of their houses, being injured, or losing their loved ones, and have to live in shelters or tents. All these factors can justify insomnia and reduced sleep quality experienced by them.

Our results showed that some demographic components such as age, education, and occupation were associated with unhealthy clusters. That is, the risk of being placed in unhealthy clusters was lower for younger participants and for the illiterate samples, while the risk of being in unhealthy clusters was higher for unemployed and students. Contrary to the results of the present study, sleep quality decreased with increasing age ^39^. However, past studies have been done in normal living conditions, while our samples were in abnormal conditions following an earthquake. The reason for being placed young people in unhealthy clusters is probably related to factors such as lack of emotion regulation skills in critical post-earthquake situations. Adults seem to be better able to adapt to their situation because of their experience, especially because of the eight-year Iraq war. In the other part, the results of the present study are in line with previous studies; the quality of sleep is poor among students^40^. This may be due to variables such as stress and anxiety, because these variables can decrease sleep quality.

This study was conducted among earthquake victims in Kermanshah, Iran. Sampling was based on voluntary participation. Therefore, results must be generalized to other populations with caution. Another limitation of the present study was the high percentage (22%) of non-responders, which can be considered in generalizing the results.

## Conclusion

Personality traits, experiential avoidance, stress, anxiety, depression, and dysfunctional beliefs and attitudes about sleep can predict clusters formed based on sleep quality and insomnia severity. Personality traits, experiential avoidance, stress, anxiety, depression, and dysfunctional beliefs and attitudes about sleep can contribute to sleep problems and reduce sleep quality in earthquake victims. Therefore, psychologists are recommended to consider the role of these variables while attempting to improve sleep quality and decrease insomnia severity in those experiencing stressful conditions such as earthquakes. Finally, people with neuroticism personality traits that have high scores in stress and depressive and anxiety disorders suffer from sleep problems after the earthquake.

## Acknowledgements

We thank all those who helped prepare this work. The authors appreciate the Sleep Disorders Research Center and Clinical Research Development Center of Imam Reza Hospital (Kermanshah University of Medical Sciences) to collaborate on preparing this project.

## Conflict of interest

None declared.

## Funding

Kermanshah University of Medical Sciences (grant number: 96640).

## Highlights

- Sleep profile is different in the earthquake victims.
- More than a third of the victims are in the pathological clusters.
- Younger age and lower education are protective factors; although unemployment and being a student are risk factors related to the pathological clusters.
- Maladaptive personality traits, psychological distress, experiential avoidance, and dysfunctional beliefs about sleep are related to the pathological clusters.
